# Contrasting bacterial and archaeal distributions reflecting different geochemical processes in a sediment core from the Pearl River Estuary

**DOI:** 10.1186/s13568-020-0950-y

**Published:** 2020-01-22

**Authors:** Wenxiu Wang, Jianchang Tao, Haodong Liu, Penghui Li, Songze Chen, Peng Wang, Chuanlun Zhang

**Affiliations:** 1 State Key Laboratory of Marine Geology, Tongji University, Shanghai, China; 2 Shenzhen Key Laboratory of Marine Archaea Geo-Omics, Department of Ocean Science and Engineering, Southern University of Science and Technology, Shenzhen, China; 3 Laboratory for Marine Geology, Qingdao National Laboratory for Marine Science and Technology, Qingdao, China

**Keywords:** Bacterial and archaeal communities, Depth variability, Geochemical cycles, Pearl River estuarine sediments

## Abstract

Microbial community structure and metabolic activities have profound impacts on biogeochemical processes in marine sediments. Functional bacteria such as nitrate- and sulfate-reducing bacteria respond to redox gradients by coupling specific reactions amenable to relevant energy metabolisms. However, similar functional patterns have not been observed for sedimentary archaea (except for anaerobic methanotrophs and methanogens). We coupled taxonomic composition with comprehensive geochemical species to investigate the participation of distinct bacteria and archaea in sedimentary geochemical cycles in a sediment core (300 cm) from Pearl River Estuary (PRE). Geochemical properties (NO_3_^−^, dissolved Mn and Fe, SO_4_^2+^, NH_4_^+^; dissolved inorganic carbon (DIC), δ^13^C_DIC_, dissolved organic carbon (DOC), total organic carbon (TOC), δ^13^C_TOC_, and fluorescent dissolved organic matter (FDOM)) exhibited strong depth variability of different trends. Bacterial 16S rRNA- and *dsrB* gene abundance decreased sharply with depth while archaeal and bathyarchaeotal 16S rRNA gene copies were relatively constant. This resulted in an increase in relative abundance of archaea from surface (11.6%) to bottom (42.8%). Network analysis showed that bacterial groups of *Desulfobacterales*, *Syntrophobacterales* and *Gammaproteobacteria* were significantly (P < 0.0001) associated with SO_4_^2−^ and dissolved Mn while archaeal groups of *Bathyarchaeota*, Group C3 and Marine Benthic Group D (MBGD) showed close positive correlations (P < 0.0001) with NH_4_^+^, δ^13^C_TOC_ values and humic-like FDOM. Our study suggested that these bacterial groups dominated in redox processes relevant to sulfate or metal oxides, while the archaeal groups are more like to degrade recalcitrant organic compounds in anaerobic sediments.

## Introduction

Estuarine ecosystems are highly dynamic and one of the most productive environments due to enriched carbon and nutrients (Cai 2011). Estuarine sediments usually harbor considerable microbial biomass and activities associated with the remineralization of sedimentary organic carbon, which has a significant impact on biogeochemical cycles (Burdige 2011). Geochemically, the depth sequence of oxidants used in the mineralization of organic matter is O_2_, NO_3_^−^, Mn(IV), Fe(III), SO_4_^2−^, and CO_2_ (Froelich et al. 1979); biologically, nitrate-, iron and manganese oxides- and sulfate-reducing bacteria are known to respond to redox gradients in the oxygen-depleted environments. Accordingly, bacterial community compositions can be linked to the vertical succession of predominant terminal electron acceptors (Algora et al. 2015; Walsh et al. 2016).

Archaeal groups responding to redox gradients in the sedimentary environments can be broadly characterized, with Marine Group I (ammonia-oxidizing archaea) occupying the surface or shallow oxic environments and Woesearchaeota perhaps the deeper anoxic environments (Dang et al. 2013; Nunoura et al. 2018). Although Bathyarchaeota and other uncultivated archaea are frequently found in anoxic sediments (Lazar et al. 2015; Zhou et al. 2018), only methanogens and methane-oxidizing archaea (ANMEs) can surely be defined as strict anaerobes, which are commonly present at or below the sulfate-methane transition zone (SMTZ) (Thauer et al. 2008). Most of the sedimentary archaea have resisted being brought into pure culture, which has kept their physiology and biogeochemical roles elusive.

Isotopic evidence from Peru margin has shown that the dominating sedimentary archaea may be heterotrophic (Biddle et al. 2006). *Bathyarchaeota* and Marine Benthic Group D (MBGD) exhibit high abundances in subsurface sediments and prefer low sulfide, high TOC conditions (Kubo et al. 2012; Lazar et al. 2015; Pan et al. 2019) or hydrate-bearing marine sediments (Inagaki et al. 2006), but their involvement in methane production or oxidation is controversial (Biddle et al. 2006; Kubo et al. 2012). Recent studies based on genomic analysis revealed a variety of metabolisms in uncultured archaea. These are exemplified by sulfur- or sulfate reduction in *Hadesarchaeota*, *Thorarchaeota*, *Theionarchaea* and *Bathyarchaeota* (Baker et al. 2016; Lazar et al. 2017; Seitz et al. 2016; Zhang et al. 2016), nitrate- or nitrite reduction in *Hadesarchaeota*, *Bathyarchaeota* and MBGD (Baker et al. 2016; Lazar et al. 2016; Zhou et al. 2018), methane and/or short alkane metabolism in *Bathyarchaeota*, *Verstratearchaeota*, *Hadesarchaeota*, *Helarchaeota* and other TACK superphylum archaea (Evans et al. 2015; Seitz et al. 2019; Vanwonterghem et al. 2016; Wang et al. 2019), and detrital protein degradation and acetogenesis in most of anaerobic archaea (He et al. 2016; Lazar et al. 2017; Lloyd et al. 2013). *Lokiarchaeota* may be capable of iron reduction in sediments of the Arctic mid-ocean ridge (Jorgensen et al. 2012; Sousa et al. 2016). A lokiarchaeon (*Candidatus Promethearchaeum syntrophicum* strain MK-D1) was firstly cultured from methane seep sediments, which was able to utilize amino acids in syntrophy with *Methanogenium* (Imachi et al. 2019).

To date, most of these predicted metabolisms have yet to be proven except three studies of enrichment and pure cultures (Imachi et al. 2019; Yu et al. 2018; Vanwonterghem et al. 2016). Hence, more investigations are required to better understand the geochemical functions of uncultured archaea.

In this study, geochemical methods were used for quantifying and characterizing different chemical species, and molecular techniques with quantitative PCR (qPCR) and sequencing for different bacterial and archaeal populations along the depth of a 300-cm sediment core from eutrophic Pearl River Estuary (PRE). Large differences in distributional patterns of bacterial and archaeal groups as well as the functional genes were found along the environmental profile, allowing the differentiation of their potential roles involved in geochemical processes.

## Materials and methods

### Sediment sampling

A 300-cm gravity core was collected at 21-m water depth in PRE (22.1315 N, 113.8055 E) in October 2017. Sampling of pore water and sediment for geochemical and microbial analyses was conducted onboard the ship as quickly as possible. The core was sectioned into 5-cm intervals for 0–100 cm and into 10-cm intervals for 100–300 cm below sediment surface. DNA samples were snap-freezed in liquid nitrogen before storage at − 80 °C. Pore water was extracted with Rhizon samplers (Dickens et al. 2007). For sulfate measurements, 2-ml pore water was acidified with 50% HCl to remove volatile sulfur compounds and then stored at 4 °C. For total dissolved Mn and Fe measurements, 2-ml pore water was acidified with 65% HNO_3_ and then stored at 4 °C. Pore water samples and sediment samples were also stored in a − 20 °C freezer; the former samples were used for analyses of dissolved inorganic carbon (DIC), dissolved organic carbon (DOC), fluorescent dissolved organic matter (FDOM) and nutrients, and the latter for total organic carbon (TOC).

### Geochemical analysis

Sulfate concentrations were diluted (1:500) and measured on an ionic chromatograph (Dionex ICS-1500, USA). Total dissolved Mn and Fe levels were determined with an inductively coupled plasma atomic emission spectrometry. NO_3_^−^ and NH_4_^+^ were analyzed using an autoanalyzer (BRAN and LUEBBE AA3, Germany). Concentrations and carbon isotopic compositions of DIC were analyzed on a continuous-flow isotope ratio mass spectrometer (MAT 253, Gas Bench). The precision based on duplicate analysis was ± 0.2‰ for δ^13^C_DIC_. Sediments for TOC analyses were freeze-dried and ground. TOC content was determined on an elemental analyzer (Vaio EL Cube, Germany) after acid dissolution of carbonates from an aliquot of dried sediment powder. Stable carbon isotopes of TOC (δ^13^C_TOC_) were measured using a Thermo Science Delta Plus isotope ratio mass spectrometer connected on-line to a Carlo Erba Instruments Flash 1112 elemental analyzer. The analytical precision was ± 0.1% for TOC content and ± 0.2‰ for δ^13^C_TOC_.

### Spectroscopic analysis and parallel factor analysis (PARAFAC) for DOM

Spectroscopic analysis of DOM samples (GSD-110 and GSD-270 were not included) was conducted on a fluorescence spectrometer (Jobin–Yvon Horiba Aqualog-800-C, Horiba Instruments). Fluorescence excitation-emission matrix (EEM) spectra were generated from 240 to 450 nm at 2 nm increments for excitation (Ex) wavelengths, and from 245.90 to 829.35 nm at 1.17 nm increments for emission (Em) wavelengths. All the sample spectra were normalized to Raman peak area after corrected with the ultrapure water EEM spectra, and reported in Raman unit (R.U.) (Murphy 2011). Finally, parallel factor analysis (PARAFAC) was carried out in MATLAB 2013 (Mathworks, USA) with the DOM Fluor toolbox (http://www.models.life.ku.dk).

### DNA extraction, qPCR and amplicon sequencing

DNA was extracted from 0.2 to 0.3 g of sediment (wet weight) using a PowderSoil DNA Isolation kit (Mo Bio) according to the instruction and stored at − 20 °C until further analyses. Targeted genes for qPCR analyses included 16S rRNA genes for bacteria, archaea, *Bathyarchaeota*; *dsrB* gene for sulfate-reducing bacteria (SRB), *nirS* gene for denitrifying bacteria; *mcrA* gene for methanotrophic and methanogenic archaea. The primers and standards used in this study are shown in Additional file 1: Table S1. The qPCR analyses were performed on a QuantStudio 5 Real-Time PCR System and the reaction volume was 10 μl: 5 μl of SYBR Premix Ex Taq™ II (TaKaRa), 0.4 μM of each primer, 0.1 μl of 1% bovine serum albumin (BSA), 0.2 μl of ROXII (TaKaRa), 2.9 μl of ddH_2_O and 1 μl of template DNA. The amplification conditions were as follows: 30 s denaturation at 95 °C and 40 cycles of denaturation at 95 °C for 5 s, annealing at 55 °C for 45 s and extension at 72 °C for 1 min. The linear correlation coefficients (R^2^) ranged from 0.99 to 1.00, and the amplification efficiencies were between 90 and 110%.

The V4 region of the prokaryotic 16S rRNA gene was PCR amplified with universal prokaryotic primers 515FB (GTGYCAGCMGCCGCGGTAA) and 806RB (GGACTACNVGGGTWTCTAAT) (Caporaso et al. 2012). The 50 μl of PCR mixture consisted of 25 μl of 2× Premix Taq DNA polymerase (TaKaRa), 0.2 mM of each primer, 20 μl of ddH_2_O, and 3 μl of template DNA. Procedures for the PCR were as follows: 30 s denaturation at 94 °C, 30 s annealing at 58 °C and 30 s extension at 72 °C, repeated for 30 cycles in a BioRad S1000 (Bio-Rad Laboratory, CA). The PCR products were pooled and purified using the EZNA Gel Extraction Kit (Omega, USA). Sequencing was conducted on the Miseq platform (2 × 250 PE, lllunina) at the Guangzhou Magigene Biotechnology (Guangzhou, China).

Raw Miseq data were analyzed using the Quantitative Insights into Microbial Ecology (Qiime2, version 2018.4) with plugins demux, DADA2 and feature-table. Features with a total abundance of less than 10 and those present in only a single sample were filtered out. Sequence Taxonomy was assigned using the Silva 128 99% Operational Taxonomic Units (OTUs) database (https://www.arb-silva.de/ngs). Shannon diversity index was calculated for alpha diversity analysis.

### Statistical analysis

Principal coordinates analysis (PCoA) was used to evaluate the compositional changes of microbial community based on Bray–Curtis distance. Species tables are normalized and square root transformed before PCoA and analysis of the similarity (ANOSIM) performed in PRIMER software package (Clarke and Gorley 2006). Linear discriminant analysis (LDA) effect size (LEfSe) (Segata et al. 2011) was used to identify microbial populations with significant difference between upper and deep layers. Spearman correlations between the relative abundance of OTUs and environmental parameters were conducted in R 3.5.0, and performed in network by Cytoscape 3.6.1 (Shannon et al. 2003). OTUs related to environmental parameters were selected to build a Maximum-likelihood (ML) phylogenetic tree with their reference sequences from different sampling sites using MEGA 7.0 (Kumar et al. 2016).

### Sequencing results and deposition

Source sequences are available in the GenBank nucleotide sequence database under the Accession number PRJNA575161.

## Results

### Geochemistry of pore water and sediments

**Fig. 1:**
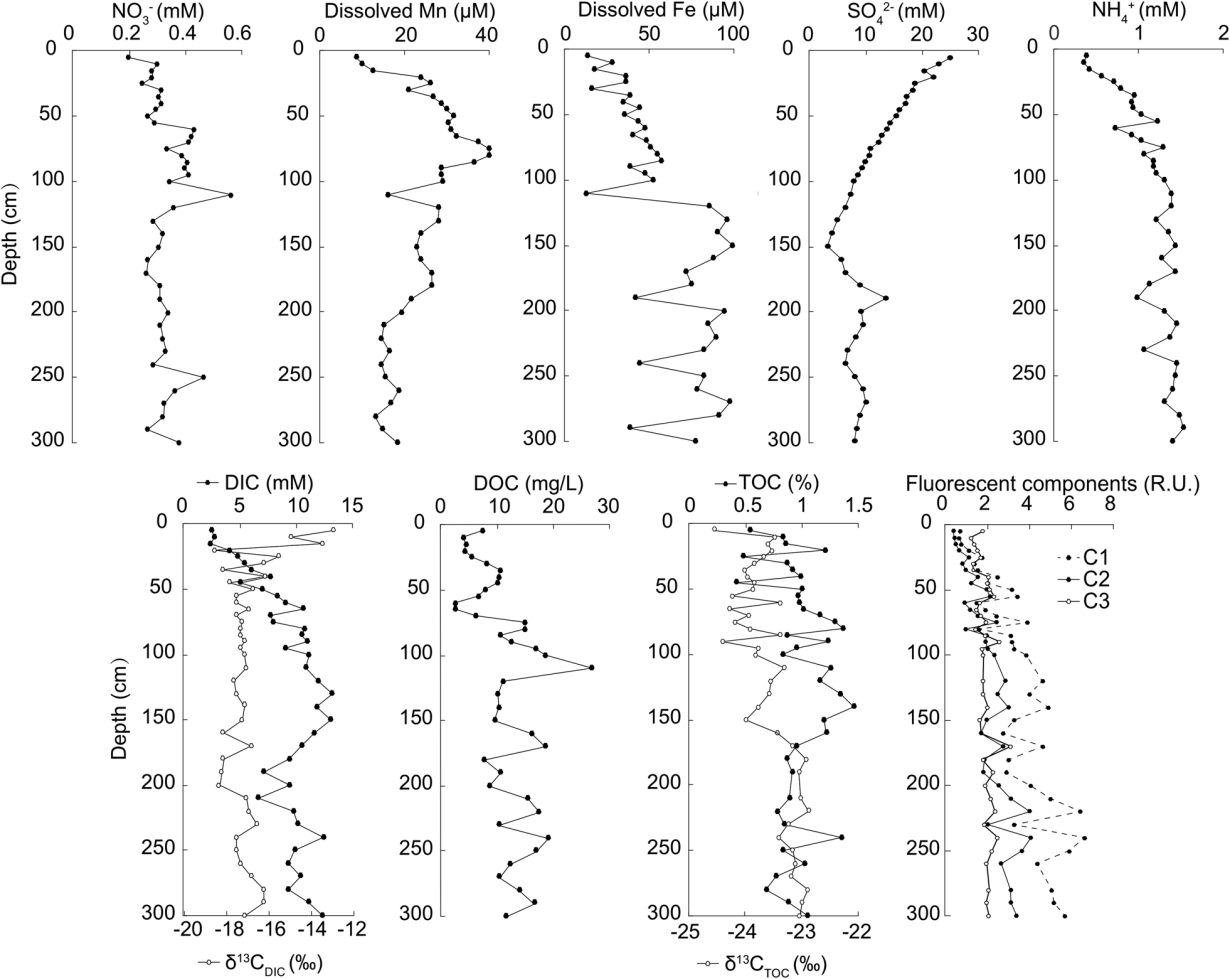
Characteristics of pore water chemistry (NO_3_^−^, NH_4_^+^, dissolved Mn, Fe, and SO_4_^2−^, DIC, DOC, and fluorescent components) and sediment total organic carbon (TOC). Collection of pore water for geochemical analyses was conducted onboard the ship as quickly as possible using Rhizon samplers

Geochemical profiles were described in the order of nitrate reduction, Mn/Fe reduction, and sulfate reduction (Fig. 1). NO_3_^−^ maintained high concentrations at all depths, making the nitrogenous zone ambiguous. Total dissolved Mn followed a typical diagenetic profile and reached maximum at 75-cm depth. Total dissolved Fe increased in the deep layers as dissolved Mn decreased, probably defining the shift from the manganous zone to the ferruginous zone. SO_4_^2−^ decreased linearly from ~ 25 to ~ 3 mM in the upper 150 cm and then increased temporarily before staying at ~ 9 mM until the bottom depth (300 cm). The drop in sulfate concentration from surface value indicated an overall occurrence of microbial sulfate reduction within the sediment core. Dissolved oxygen was not determined.

NH_4_^+^ gradually increased down-core from ~ 0.4 mM to higher than 1 mM. DIC and DOC concentrations increased downward and peaked at ~ 12 mM at 150-cm depth and ~ 27 mg/l at 110-cm depth, respectively. δ^13^C_DIC_ values decreased down-core to a range between − 18.6 and − 13.0‰. Keeling plot analysis of DIC showed δ^13^C values of around − 18.4‰ for excess DIC (Additional file 2: Fig. S1), implying that the accumulation of DIC was mainly derived from the respiration of organic matter. TOC content varied between 0.4 and 1.5% along the core. δ^13^C_TOC_ values ranged from − 24.5 to − 22.8‰ and showed difference below and above 150-cm, indicating a minor change of organic matter from marine plankton-derived to terrestrial plants-derived (Wu et al. 1999).

### PARAFAC components of DOM

Three different PARAFAC components were identified, including two humic-like components (C1 and C2) and one protein-like component (C3). Both C1 and C2 were identified as a combination of two EEM peaks (peak A and peak M for C1, peak A and peak C for C2; Additional file 3: Table S2). Peak A and peak C are depicted as terrestrially derived humic substances and peak M as the marine humic-like substances or products from microbial processes (Coble 1996; Stedmon and Markager 2005). C3 exhibiting its emission maxima at 307 nm and its excitation maxima at 274 nm, corresponded to tyrosine-like and/or protein-like substances (peak B; Additional file 3: Table S2) (Coble 1996). Overall, humic-like component C1 was the most abundant fraction (45.0%), followed by humic-like component C2 (27.7%) and protein-like component C3 (27.3%). C1, C2 and C3 varied differently from each other with increasing depth (Fig. 1). Humic-like components (C1 and C2) showed marked increases downward, while the protein-like component (C3) was relatively constant.

### Distributions of bacterial and archaeal abundances

**Fig. 2:**
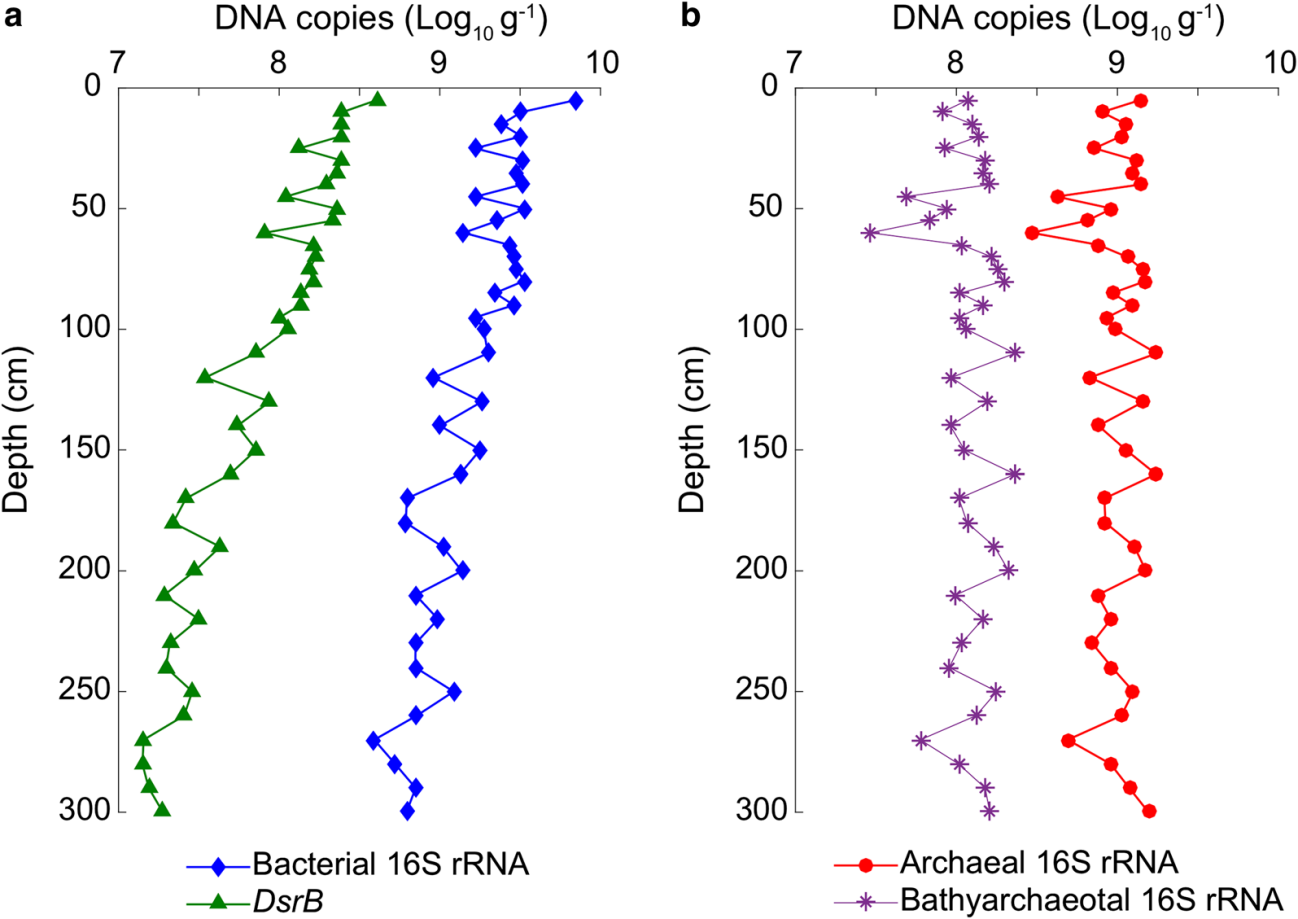
Depth-variations in microbial 16S rRNA- and *dsrB* gene copies based on qPCR. **a** Bacterial 16S rRNA- and *dsrB* genes; **b** archaeal 16S rRNA- and bathyarchaeotal 16S rRNA genes

The bacterial 16S rRNA and *dsrB* gene abundances decreased from 7.03 × 10^9^ to 3.92 × 10^8^ copies/g sediments and from 4.05 × 10^8^ copies/g sediments to 1.41 × 10^7^ copies/g sediments, respectively (Fig. 2). The bacterial 16S rRNA and *dsrB* gene copies were in the same range with those reported in the eutrophic PRE (Jiang et al. 2009). Both of them sharply declined with sediment depth. Besides, bacterial abundance was strongly, negatively correlated to δ^13^C_TOC_ (r = − 0.75, P < 0.0001), and positively correlated to the C3/(C1 + C2) ratio (r = 0.76, P < 0.0001). *DsrB* gene abundance also exhibited high correlation with SO_4_^2−^ (r = 0.82, P < 0.0001), δ^13^C_TOC_ (r = − 0.70, P < 0.0001) and the C3/(C1 + C2) ratio (r = 0.80, P < 0.0001). The abundances of *nirS* gene and *mcrA* gene were much lower than *dsrB* gene at all depths (data not shown).

Archaeal and bathyarchaeotal 16S rRNA gene abundances varied differently from that of bacteria along the depth (Fig. 2). Archaeal and bathyarchaeotal 16S rRNA gene abundances showed a slight increase down core, with the maximum of 1.75 × 10^9^ copies/g sediments at 110-cm and 2.30 × 10^8^ copies/g sediments at 110-cm and 160-cm depth, respectively. The archaea-to-bacteria abundance ratios increased from 0.19 at 5-cm depth to 2.51 at 300-cm depth, which were higher than that previous reported in PRE (Jiang et al. 2011) and in South China Sea (Yu et al. 2017).

### Distributions of the most abundant bacterial and archaeal phyla

Illumina sequencing of 38 sediment samples (GSD-250 and GSD-270 were not included) yielded a total of 2,580,094 high-quality sequences after quality control, ranging from 47,995 to 83,260 reads in each sample. Sequence numbers in each sample reduced to 38,887 after rarefaction for further analyses. Those sequences covered the diversity of microbial populations in samples as Shannon diversity indices reached stable values (Additional file 2: Fig. S2). In total, 5553 OTUs were generated on the basis of 100% sequence identity, including 4164 for bacteria, 1268 for archaea and 121 as unassigned OTUs. The bacterial and archaeal OTUs were classified into 47 and 17 phyla, respectively. Ten of 47 bacterial phyla and five of 17 archaeal phyla had relative abundances of > 1%.

**Fig. 3:**
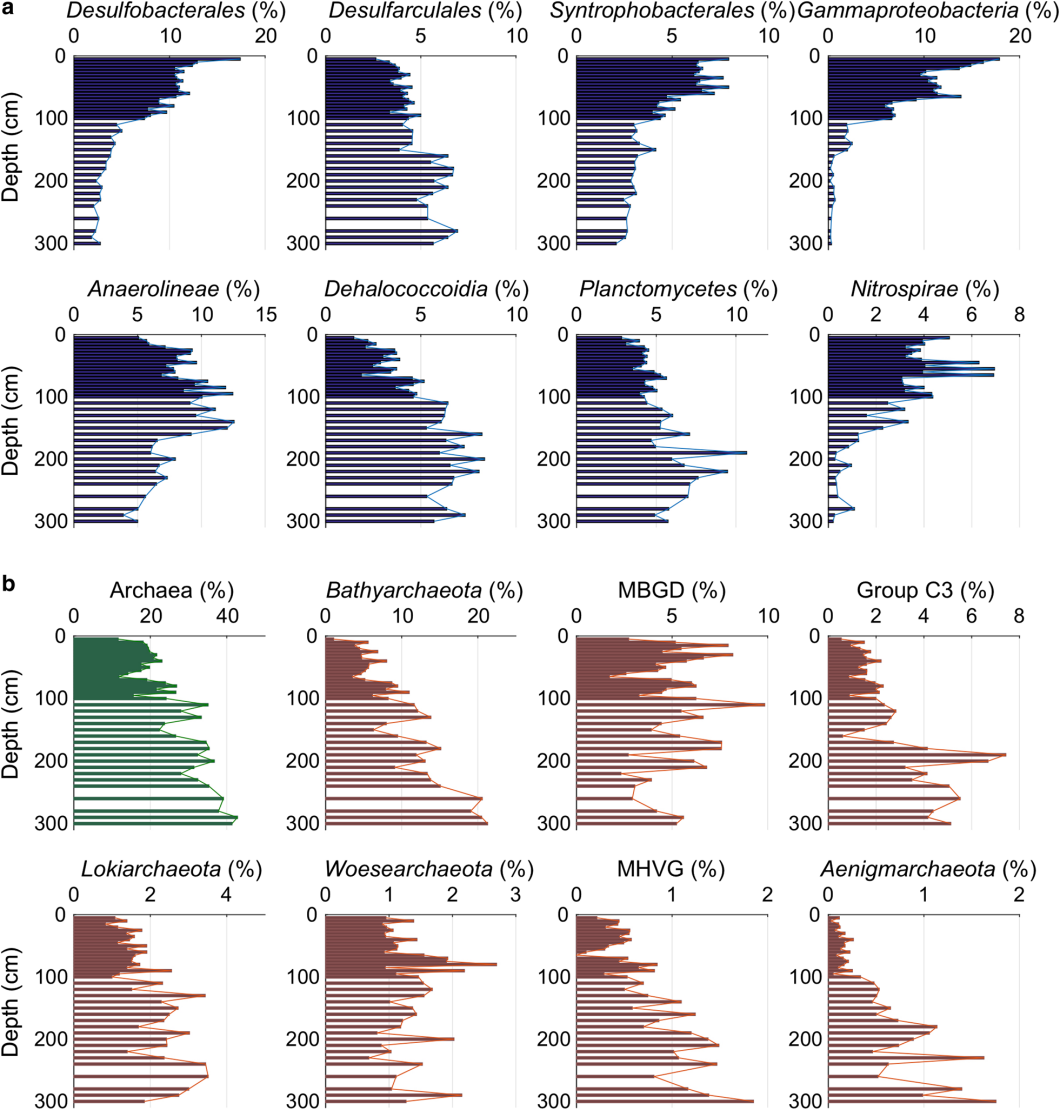
Depth distributions of relative abundances of bacteria (**a**) and archaea (**b**). Dark blue = bacterial groups, green = total archaea and dark red = different archaeal groups

*Proteobacteria*, *Chloroflexi*, *Planctomycetes* and *Nitrospirae* were the top four bacterial phyla (Fig. 3a). The great majority of Proteobacteria could be assigned to *Desulfobacterales*, *Desulfarculales*, *Syntrophobacterale*s (three orders of *Deltaproteobacteria*) and *Gammaproteobacteria*. All of them decreased with depth and exhibited significantly positive correlations with SO_4_^2−^ in pore water (r > 0.80, P < 0.0001 of all) except for *Desulfarculales* that increased slightly and showed a positive correlation with δ^13^C_TOC_ (r = 0.74, P < 0.0001). *Anaerolineae* and *Dehalococcoidia* were two major classes of *Chloroflexi*. All reads of *Anaerolineae* were affiliated to the family *Anaerolineaceae*, exhibiting a depth profile with the relative abundance decreased after reaching its maximum at 140-cm depth. The relative abundance of *Dehalococcoidia* increased down-core and showed positive correlation with NH_4_^+^ (r = 0.76, P < 0.0001). In addition, an increase in the relative abundance of *Planctomycetes* was coincided with relatively decrease of *Nitrospirae*, both of which were known to potentially involve in the nitrogen cycle (Schmid et al. 2003; Ushiki et al. 2017).

The main archaeal groups included *Bathyarchaeota*, Group C3 (affiliated to Bathy-15), MBGD (a family of *Euryarchaeota*), *Lokiarchaeota*, *Woesearchaeota*, Marine Hydrothermal Vent Group (MHVG) and *Aenigmarchaeota* (Fig. 3b). The relative abundance of *Bathyarchaeota* increased by about 20-folds (from 1.06 to 21.3%), and showed positive correlations with NH_4_^+^ and δ^13^C_TOC_ values (r = 0.68, P < 0.0001 of both). Similar depth profiles also appeared in *Lokiarchaeota*, MHVG and *Aenigmarchaeota*, while MBGD and *Woesearchaeota* seemed to be constant with depth. The relative abundance of Group C3 exhibited a depth profile with a sharp increase below 150-cm depth. In general, the relative abundance of archaea increased from 11.6 to 42.8% and showed strong positive relationship with the content of humic-like FDOM (C1 + C2) (r = 0.72, P < 0.0001).

**Fig. 4:**
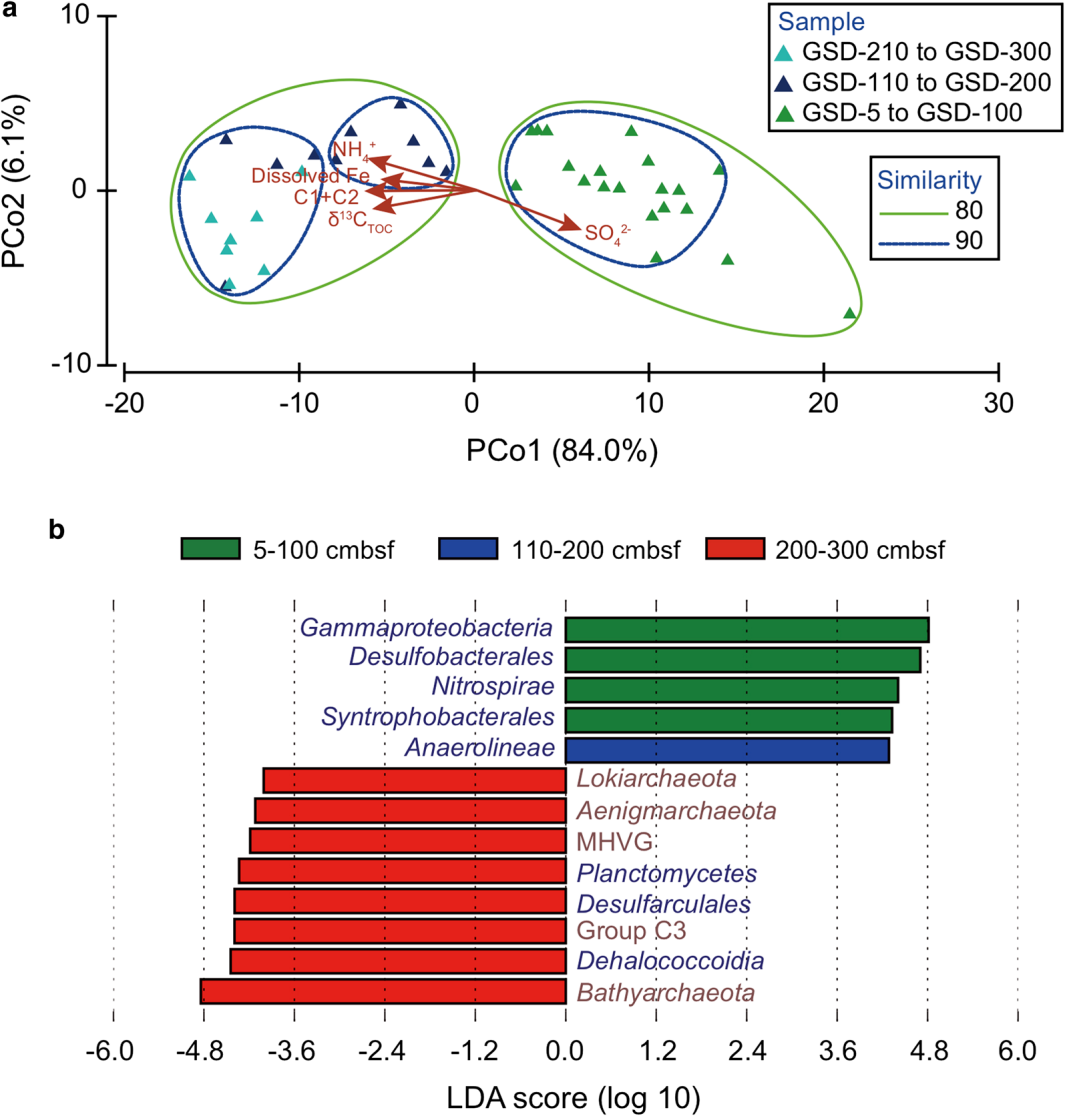
Principal coordinates analysis (PCoA) (**a**) and linear discriminant analysis (LDA) effect size (LEfSe) of microbial groups (**b**) with a LDA threshold of 3.5. In (**b**), dark red = archaeal groups and dark blue = bacterial groups

PCoA of microbial abundance showed clustering of samples along the depth (GSD-5 to GSD-100, GSD-110 to GSD-200, GSD-210 to GSD-300) (Fig. 4a). The PCo1 (PCo1 explained 84.0% of the variance) showed high correlations with SO_4_^2−^ (r = 0.72, P < 0.0001), NH_4_^+^ (r = − 0.75, P < 0.0001), dissolved Fe (r = − 0.66, P < 0.0001), δ^13^C_TOC_ values (r = − 0.72, P < 0.0001), humic-like FDOM (C1 + C2) (r = − 0.77, P < 0.0001). Next, LEfSe confirmed the tendency that most of the archaeal groups and *Dehalococcoidia*, *Desulfarculales* and *Planctomycetes* were significantly abundant in deep layers, whereas *Gammaproteobacteria*, *Desulfobacterales*, *Nitrospirae*, *Syntrophobacterales* and *Anaerolineae* were more abundant in the upper layers (Fig. 4b).

### Specific linkages between environmental variables and bacterial or archaeal species revealed by network analysis

**Fig. 5:**
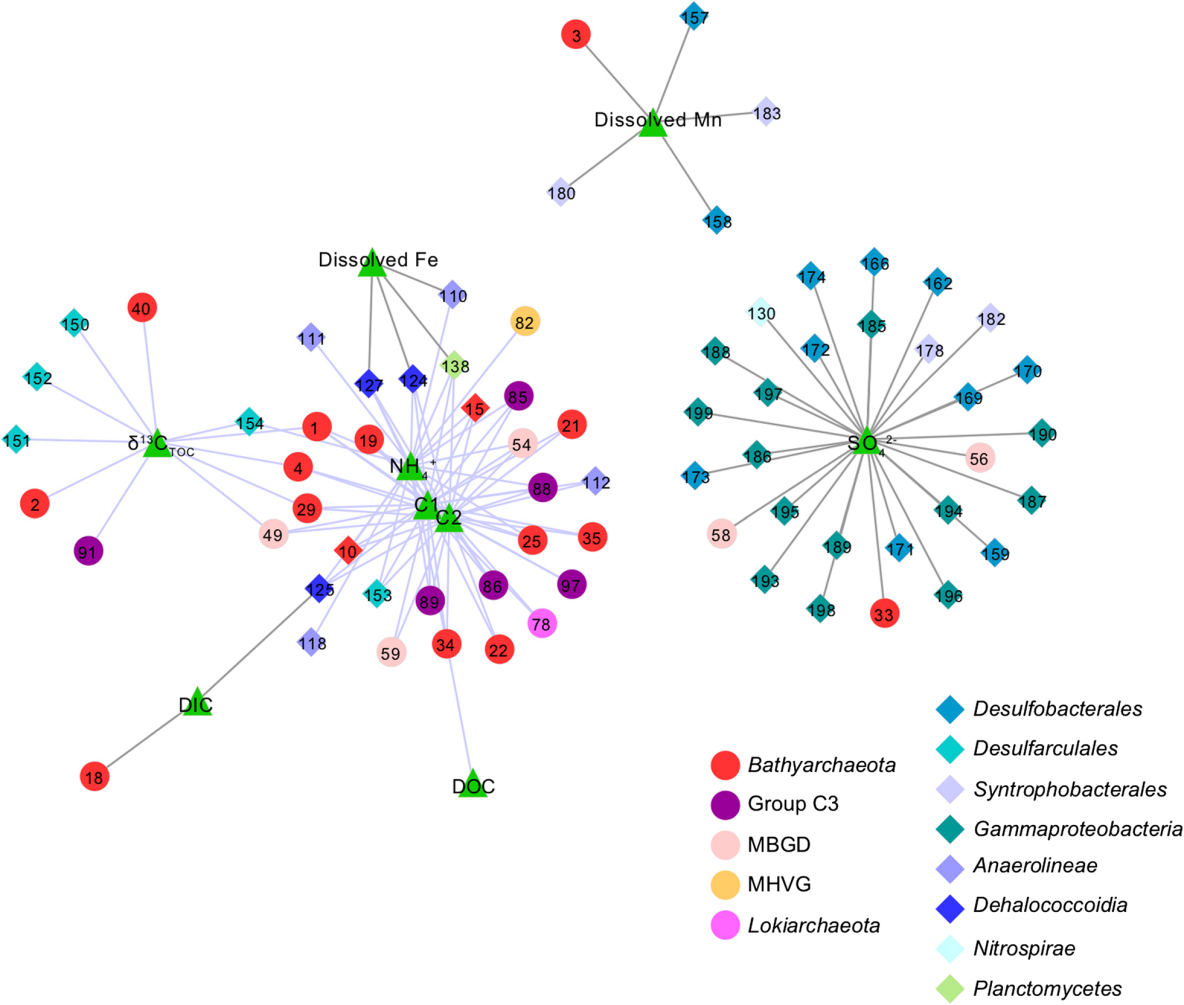
Network interactions revealed relationships between microbial and geochemical factors with a threshold of 0.65 for spearman’s coefficient and 0.0001 for P-value. Purple lines indicated positive correlations with electron donors. Gray lines indicated positive correlations with electron acceptors. Circles: Archaeal OTUs; Diamonds: Bacterial OTUs; Triangles: Geochemical factors. The numbers representing generated OTU IDs were shown in Additional file 4: Table S3

To further investigate the impacts of geochemical factors on the distribution of microbial populations, the top 100 OTUs of the eight bacterial groups and the top 100 OTUs of the seven archaeal groups (Additional file 4: Table S3) were selected to analyze their correlations with geochemical factors. These OTUs accounted for 38.4% of the total sequences. Network analysis was conducted based on positive Spearman correlation ($\rho$ > 0.65, P < 0.0001) and consisted of 79 nodes and 123 edges (Fig. 5). Geochemical parameters were roughly divided into two groups with different edge colors, grey for electron acceptors-related variables (SO_4_^2+^, NO_3_^−^, dissolved Mn and Fe, DIC, and δ^13^C_DIC_) and orange for electron donors-related variables (NH_4_^+^, DOC, TOC, δ^13^C_TOC_, C1, C2 and C3).

SO_4_^2−^ exhibited close correlations with OTUs belonging to *Syntrophobacterales*, *Desulfobacterales* and *Gammaproteobacteria*. Two OTUs of MBGD and one of *Bathyarchaeota* also correlated with SO_4_^2−^. Dissolved Mn showed positive correlations with four OTUs belonging to *Desulfobacterales*, *Syntrophbacterales* and *Bathyarchaeota*, while dissolved Fe was more closely related to OTUs that were affiliated to *Dehalococcoidia*, *Anaerolineae* and *Planctomycetes*. This result was different from the findings in San Pedro Basin (Monteverde et al. 2018) where dissolved Fe exhibited close positive correlations with proteobacterial OTUs. Bacterial or archaeal OTUs showed few direct correlations with DOC and DIC, but were closely correlated to δ^13^C_TOC_ and NH_4_^+^. Moreover, eighteen archaeal OTUs belonging to *Bathyarchaeota*, Group C3, MBGD and *Lokiarchaeota* and seven bacterial OTUs belonging to *Dehalococcoidia*, *Anaerolineae* and *Desulfarculales* were positively correlated with C1 and C2. Notably, more archaeal OTUs were related to electron donors than electron acceptors.

### Phylogenetic analysis of 16S rRNA gene

**Fig. 6:**
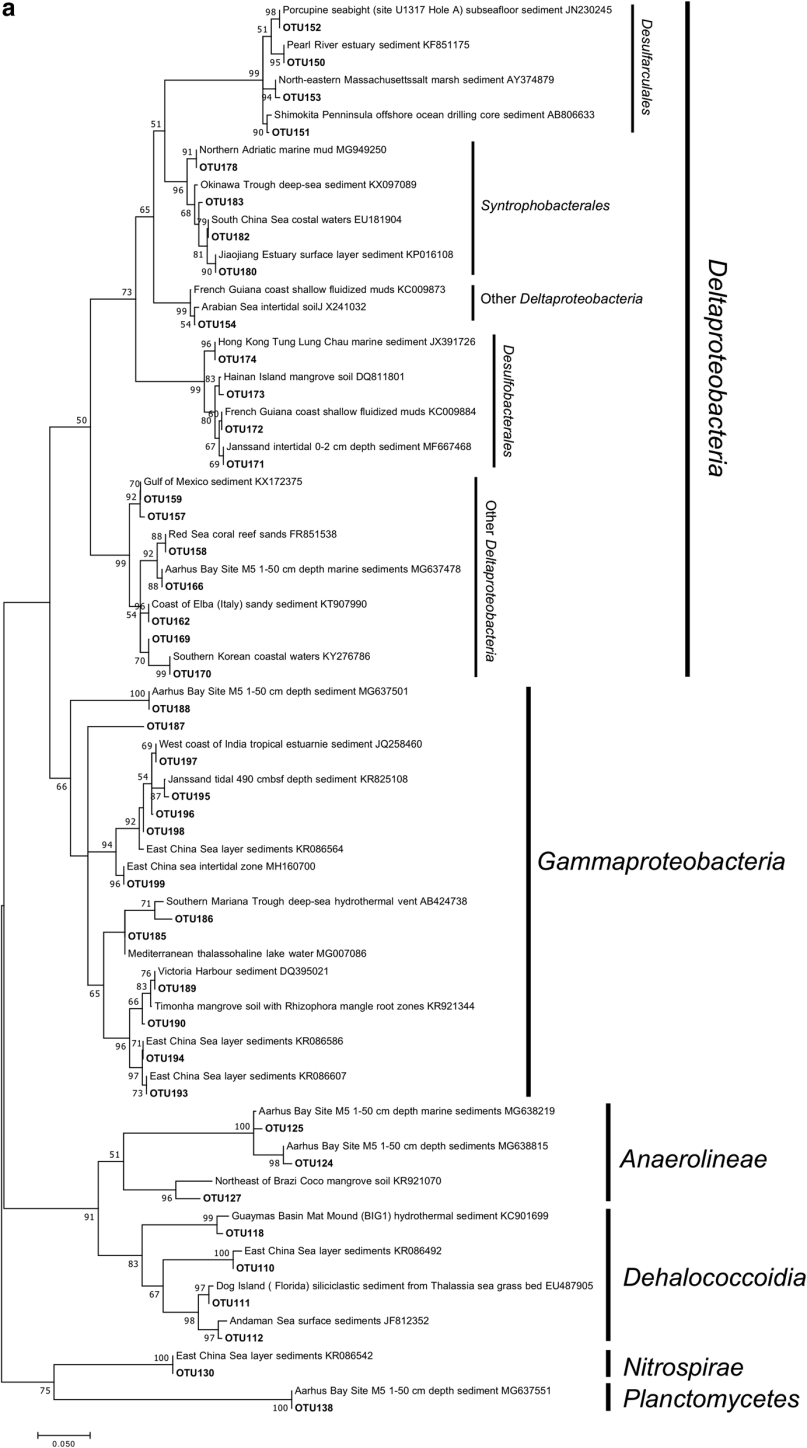

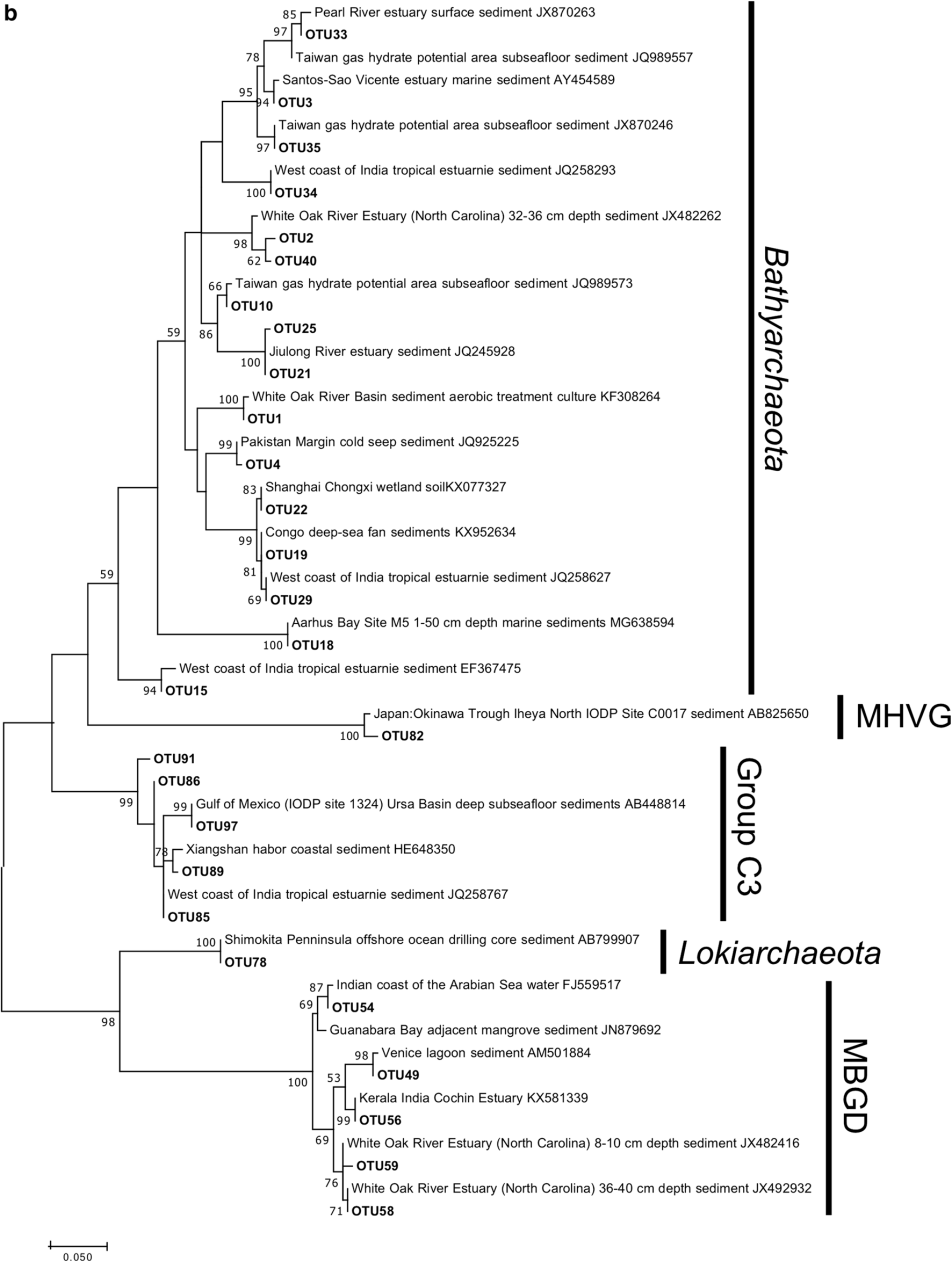
The maximum likelihood tree of 16S rRNA gene sequences showing the phylogenetic affiliations of the bacterial (**a**) and archaeal (**b**) OTUs significantly related to geochemical factors (Spearman, $\rho$ > 0.65, P < 0.0001). *MHVG* marine hydrothermal vent group. *MBGD* marine benthic group B

A total of 72 OTUs significantly related to geochemical factors (Spearman, $\rho$ > 0.65, P < 0.0001) were used for phylogenetic analysis, in which 44 belonged to bacteria (Fig. 6a) and 28 to archaea (Fig. 6b). The ML phylogenetic trees indicated that homologous sequences of the bacteria and archaea from Pearl River estuarine sediments were similar to those found in sediments in west coast of India, Aarhus Bay, White Oak River (WOR) estuary, East China Sea, Taiwan gas hydrate potential area and Shimokita Penninsula offshore. Five OTUs of *Anaerolineae*, *Planctomycetes*, *Deltaproteobacteria* and *Gammaproteobacteria* and one of *Bathyarchaeota* were similar to sequences derived from sulfate reduction zone (SRZ) of sediments from Aarhus Bay (Starnawski et al. 2017). Two OTUs of MBGD and two of *Bathyarchaeota* exhibited high affinity to that from SRZ or SMTZ of sediments from WOR (Lazar et al. 2015) or anaerobic cultivation of sediments from WOR (Gagen et al. 2013). In addition, three OTUs of *Bathyarchaeota* were similar to those reported in methanogenic zone of hydrate-bearing environment (Lai et al. 2011).

## Discussion

Though uncultured archaea have been reported widely from marine sediments (Inagaki et al. 2006; Jorgensen et al. 2012; Kubo et al. 2012; Lazar et al. 2015), their geochemical functions remain unclear. In this study, depth distributions of archaeal groups in absolute and relative abundances were compared to bacterial groups, and the differences may indicate their distinct roles in geochemical cycles.

Organiclastic sulfate reduction appeared to be the main remineralization process in the sediment core, overlapped by manganese oxide reduction above 100-cm depth and iron oxide reduction in the sediments below 100-cm depth. The total bacterial and SRB abundances exponentially decreased with sediment depth, corresponding to the transition from oxic state to anoxic state and to the reduced available substrates, which was consistent with universal distributions (Kallmeyer et al. 2012; Roy et al. 2012). Microbial abundance was significantly correlated to TOC content in sediment cores from the South China Sea, Peru margin, Nankai trough, Black sea and Equatorial Pacific (Lipp et al. 2008; Yu et al. 2017). The source and composition of organic matter may also affect the abundance of benthic bacteria (Qiao et al. 2018). In this study, higher bacterial abundance in the upper layers may benefit from terrestrial plants-derived (lower δ^13^C_TOC_ values) and protein-like organic matter (higher C3/(C1 + C2) ratio). The abundance of SRB was significantly affected by SO_4_^2−^ in pore water (r = 0.82, P < 0.0001) as expected. No significant correlation was found between archaeal abundance and measured geochemical parameters. Archaeal groups may not depend on availabilities of labile organic matter or sulfate, which distinguished their vertical variability from that of bacteria (Fig. 2).

Organic matter availability coupled with metabolism of Fe/Mn and sulfate reduction can shape bacterial community patterns in various ways, resulting in different depth profiles of bacterial groups (Figs. 4b, 5). *Desulfobacterales*, *Syntrophobacterales* and *Gammaproteobacteria* positively corresponded to the sulfate pore-water profile, implying their active roles in the sulfur cycle (Oni et al. 2015a; Orsi et al. 2013). On the other hand, *Deltaproteobacteria*, *Chloroflexi* and *Planctomycetes* appeared to relate to metal oxides-reducing metabolisms (Fig. 5).

Bacterial groups such as *Anaerolineaceae*, *Dehalococcoidia* and *Desulfarculales* have been linked to the degradation of recalcitrant organic compounds (Oni et al. 2015b). They were mainly present in the deep layers in previous studies (Monteverde et al. 2018; Qiao et al. 2018) and showed positive correlations with NH_4_^+^, δ^13^C_TOC_ values and humic-like FDOM in this study (Fig. 5). *Anaerolineaceae* were capable of degrading alkanes (Liang et al. 2015) under methanogenic conditions and members of the *Dehalococcoidia* or *Desulfarculales* were able of growing via diverse organohalide or aromatic hydrocarbons (An and Picardal 2014; Poritz et al. 2015). Distributional patterns of *Bathyarchaeota*, Group C3, *Lokiarchaeota*, MBGD and MHVG were similar to those of *Anaerolineaceae*, *Dehalococcoidia* and *Desulfarculales* (Figs. 4b, 5), suggesting that these organisms may be important for degradation of recalcitrant organic matter as well. Specifically, most of them showed preferences on marine-algae derived organic matter (higher δ^13^C_TOC_ values) and humic-like FDOM (C1 and C2) (Fig. 5). Closely matched queries of C1 and C2 in OpenFluor database were identified from the dark ocean, Artic seawater and sedimentary pore water (Catala et al. 2015; Chen et al. 2016, 2018). These components may accumulate from degradation of organic matter (Chen et al. 2016) and appear to be refractory with turnover times of more than 400 years (Catala et al. 2015). Nevertheless, humic-like FDOM with high molecular weight and highly aromatic can also be bioavailable (Tfaily et al. 2018). In this study, the enriched refractory humic-like FDOM in deep layers was concurrent with the increases of archaeal relative abundance as well as Bathyarchaeal biomass.

*Bathyarchaeota*, Group C3 and MBGD have the potential in degradation of various substrates such as detrital protein, polymeric carbohydrates and fatty acids/aromatic compounds as well as acetogenesis or methane/short alkane metabolisms (Evans et al. 2015; Lazar et al. 2016; Lloyd et al. 2013; Meng et al. 2014; Wang et al. 2019; Zhou et al. 2018). It has been shown that *Bathyarchaeota* can grow on lignin as an energy source through enrichment cultivation (Yu et al. 2018). Briefly, the archaeal groups, possibly similar to *Anaerolineaceae*, *Dehalococcoidia* and *Desulfarculales*, appear to use various surviving strategies in low-energy deep layers and grow on complicated organic compounds.

Overall, our study demonstrated that functional bacterial groups (e.g., SRB) dominate in redox processes in the labile-carbon-rich upper layers such as metal oxides reduction zones and sulfate reduction zones, while archaeal groups become more competitive in deep layers involved in degradation of recalcitrant organic compounds in the anaerobic remineralization processes (excluding methane-related metabolism). In organic-carbon-rich environments, bacteria can maximize the energy availability with diverse metabolic capacities (Valentine 2007), allowing them to use a wide range of electron acceptors (NO_3_^−^, metal oxides and SO_4_^2−^). *Gamma*- and *Deltaproteobacteria* tend to be most abundant in the upper layers and correlated with sulfate concentrations (Oni et al. 2015a). As labile substrates consumed, archaeal and bacterial groups able to degrade recalcitrant compound become dominant in deep layers (Oni et al. 2015b), possibly relying on fermentation and acetogenesis to survive in deep subsurface (He et al. 2016; Lazar et al. 2016).

In summary, the microbial community structure showed depth variations in the Pearl River estuarine sediments, which may be coupled with profiles of SO_4_^2+^, NH_4_^+^, dissolved Fe, δ^13^C_TOC_, or humic-like FDOM. Bacterial and archaeal populations showed different distributional patterns in terms of relative abundance and absolute abundance. Bacterial groups including *Desulfobacterales*, *Syntrophobacterales* and *Gammaproteobacteria* showed strong positive correlations to SO_4_^2−^, and dominated in remineralization of possibly labile organic matter in the upper layers. *Chloroflexi* and most archaeal populations preferred deep layers and showed close positive correlations with NH_4_^+^, δ^13^C_TOC_ and humic-like FDOM, suggesting their participation in degradation of recalcitrant organic matter. Archaea exhibited a weaker response to electron acceptor gradients, but a better use of humic-like FDOM. In future research, detailed analyses of organic matter composition and enrichment of archaeal species in marine sediments would be necessary to delineate the processes of organic matter utilization in archaea. This study enhances our understanding of the distribution of microbial populations and offers clues for uncovering the roles of bacteria and archaea in biogeochemical cycles in the sediments of the estuarine environment.

## Supplementary information

**Additional file 1: Table S1.** Primer used in qPCR amplification.

**Additional file 2: Fig. S1.** Keeling plot of δ^13^C_DIC_ vs. 1/DIC from pore water analysis. **Fig. S2.** Shannon diversity index curves of sediment core from PRE. Each curve represents a sample (total 38 samples) from the core.

**Additional file 3: Table S2.** Fluorescence spectral characteristics of three kinds of components.

**Additional file 4: Table S3.** Annotations of top 100 OTUs belonging to archaeal and bacterial groups, respectively.
