# Supplementary material for: Contrasting bacterial and archaeal distributions reflecting different geochemical processes in a sediment core from the Pearl River Estuary
Source: AMB Express. 2020 Jan 22;10:16. doi: 10.1186/s13568-020-0950-y (PMC6975606; doi:10.1186/s13568-020-0950-y)
Supplement: Supplementary file 2 — Additional file 2: Fig. S1. Keeling plot of δ13CDIC vs. 1/DIC from pore water analysis. Fig. S2. Shannon diversity index curves of sediment core from PRE. Each curve represents a sample (total 38 samples) from the core. [file 13568_2020_950_MOESM2_ESM.docx]

**Submitted to *AMB Express***

**Contrasting bacterial and archaeal distributions reflecting different geochemical processes in a sediment core from the Pearl River Estuary**

**Wenxiu Wang^1^, Jianchang Tao^2^, Haodong Liu^2^, Penghui Li^2^, Songze Chen^1^, Peng Wang^1*^, Chuanlun Zhang^2, 3*^**

^1^State Key Laboratory of Marine Geology, Tongji University, Shanghai, China

^2^Shenzhen Key Laboratory of Marine Archaea Geo-Omics, Department of Ocean Science and Engineering, Southern University of Science and Technology, Shenzhen, China

^3^Laboratory for Marine Geology, Qingdao National Laboratory for Marine Science and Technology, Qingdao, China

*** Correspondence:**

Peng Wang (pengwang@tongji.edu.cn)

Chuanlun L. Zhang (zhangcl@sustech.edu.cn)

Fig. S1. Keeling plot of δ^13^C_DIC_ vs. 1/DIC from pore water analysis.

Fig. S2. Shannon diversity index curves of sediment core from PRE. Each curve represents a sample (total 38 samples) from the core.
